# Citryl-Imino-Chitosan Xerogels as Promising Materials for Mercury Recovery from Waste Waters

**DOI:** 10.3390/polym16010019

**Published:** 2023-12-20

**Authors:** Daniela Ailincai, Bianca Iustina Andreica

**Affiliations:** 1 Petru Poni Institute of Macromolecular Chemistry, Grigore Ghica Voda Alley, 41A, 700487 Iasi, Romania; 2 The Research Institute of the University of Bucharest (ICUB), 90 Sos. Panduri, 050663 Bucharest, Romania

**Keywords:** hydrogels, imine, supramolecular arrangement, mercury recovery

## Abstract

The present study reported the obtention of xerogels based on chitosan and citral and their use as materials for mercury ion recovery from aqueous solutions, this being a serious problem related to the environment. The systems were prepared by the acid condensation of chitosan with citral, followed by the lyophilization of the resulting hydrogels, in order to obtain highly porous solid materials. The structural, morphological and supramolecular characterization of the systems was performed using ^1^H-NMR and FTIR spectroscopy, scanning electron microscopy and wide-angle X-ray diffraction. The ability of the obtained materials to be used for the recovery of mercury from aqueous solutions revealed the high potential of the xerogels to be used in this sense, the analysis of the materials post mercury absorption experiments revealing that this ability is predominantly conferred by the imine linkages which act as coordinating moieties for mercury ions.

## 1. Introduction

Heavy metal pollution is a significant problem that arises from the release of high concentrations of heavy metals into the environment, which is toxic to living organisms at elevated levels [1,2,3]. Common heavy metals of concern in pollution include lead, mercury, cadmium, arsenic, chromium, and nickel. The impact against the environment is manifested as soil, water and air contamination, and the exposure of the human body to these metals, even at low levels, can have severe health consequences [2]. The most significant route of exposure for humans is represented by the consumption of contaminated food and water [4]. Many countries have established regulations and standards to control the release of heavy metals into the environment, which involve monitoring emissions and enforcing penalties for non-compliance [5,6]. Moreover, health agencies such as Food and Drug Administration (FDA), World Health Organization (WHO) or Centre for Disease Control (CDC) included heavy metals on the list of carcinogenic compounds due to their extremely dangerous effect on human health [2,7]. 

Among these metals, mercury was placed on a priority monitoring list by the international agencies due to its ability to sublimate, contaminating the air and further accumulating into water and soil, entering and impacting in a significant way the ecosystems [8]. According to WHO, mercury and mercury-containing compounds present high toxicity, affecting the central and peripheral nervous systems, lungs and kidneys, eyes, skin and the gastrointestinal tract [9]. The symptoms for mercury poison include insomnia, memory loss, headaches, cognitive and motor disfunctions [10]. Because of that, the recommended limits for mercury are established by the authorities at very low values, at 0.1 µg/g for soil and 6 µL/L for water [2,11]. 

In this context, the researchers’ attention was focused on developing new materials able to retain mercury from water and soil, in this manner keeping the mercury levels within the limits imposed by the law [12,13,14,15,16,17,18,19]. Moreover, in order to minimize the environmental impact and maximize the positive effect of these materials, their obtention from renewable resources is highly recommended [20]. Hence, biopolymers represent a great workbench for the development of new materials with the ability to remove mercury, including polysaccharides which, due to their capacity to create physical and electrostatic forces, are considered excellent sorbents for heavy metals, with the potential for improvement through the introduction of coordinating moieties [21,22,23,24,25,26,27]. Among the materials designed and used in this sense, hydrogels and xerogels are considered the best choice due to their porous structure, which increases the surface area and enhances the contact between the hydrogel and the metal ions in the solution, improving recovery efficiency [28,29,30].

In light of this data, the present study aimed to obtain materials in the form of hydrogels for the recovery of mercury from an aqueous solution. The hydrogels were formed from two environmentally friendly and safe reagents: chitosan and citral, both of them with natural origin [31,32]. Chitosan was chosen for the study due to its chemical structure, containing amino and hydroxyl functional groups that are able to form complexes with metal ions, including mercury [33] and also due to its well-known biological properties [34]. Citral, on the other hand, was chosen due to its strong hydrophobic character, which may increase the stability of the hydrogels in acidic environment which is specific to mercury salts solutions. Moreover, citral moieties were linked to the chitosan backbone by imine linkages, which are well known in the literature as coordinating moieties for mercury ions, which improve the efficiency of mercury recovery [12,16,35,36]. Last but not least, the designed materials were presented in the form of hydrogels, which lead to highly porous materials after the removal of water, with high surface area and therefore high ability to absorb water and mercury ions from waste water. 

## 2. Materials and Methods

### 2.1. Materials

Chitosan (147 kDa, DD = 82%), citral (95%), acetic acid, sodium acetate trihydrate, mercury acetate (99%), potassium acetate (98%), calcium acetate hydrate (99%), barium acetate (99%), manganese II acetate tetrahydrate (98%), cobalt II acetate tetrahydrate (99%), cadmium II acetate dehydrate (99%), chromium III acetate (99%), copper II acetate hydrate (98%), lead II acetate trihydrate (99%), nickel acetate tetrahydrate (98%), zinc II acetate dehydrate (99%) and ethanol were purchased from Sigma Aldrich Co. St. Louis, MO, USA and used as received. 

### 2.2. The Obtaining of the Hydrogels

**Scheme 1:**
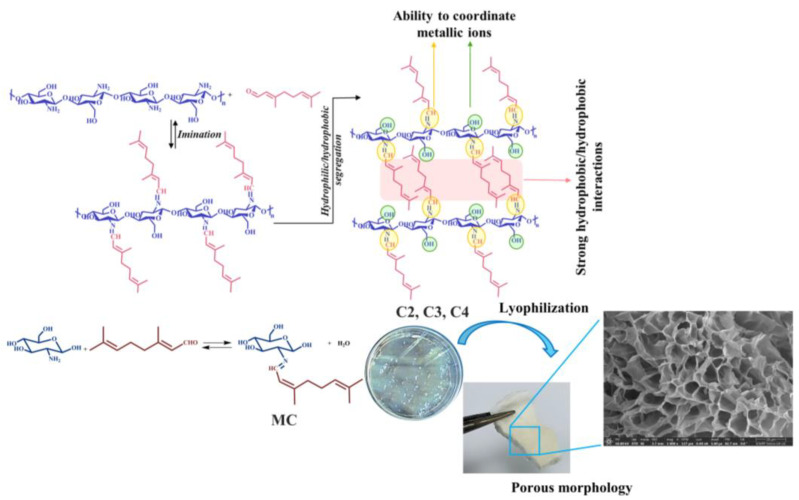
Synthetic strategy for the obtention of the hydrogels and xerogels proposed for mercury recovery and of the model compound.

Three hydrogels were prepared by the reaction of chitosan with citral in three molar ratios of their functionalities (amino group of chitosan and aldehyde group of citral), 2/1, 3/1 and 4/1 (Scheme 1, Table 1). Hydrogels were prepared by the acid condensation reaction according to the following experimental protocol: a 15 mL chitosan solution in acidic water (0.7% *v*/*v* acetic acid) was heated under magnetic stirring. When the chitosan solution reached 55 °C, a solution of citral in ethanol (1% *w*/*v*) was added dropwise. Hydrogelation occurred very fast, in less than 5 min for the 2/1 ratio, in approx. 30 min for the 3/1 ratio, and in approx. 3 h for the 4/1 ratio. The samples were noted with C followed by a number which corresponds to the molar ratio between amino and aldehyde functionalities. C represents the reference sample, which is chitosan without aldehyde. After sample preparation, the hydrogels were kept uncovered for the evaporation of ethanol used for aldehyde solubilization. 

### 2.3. The Obtaining of the Model Compound (MC)

The model compound was synthesized according to a previously described protocol, slightly improved in order to increase reaction yield [37,38]. To a solution of 100 mg glucosamine hydrochloride (0.463 mmol) in a 1.5 mL methanol, sodium hydroxide (0.463 mmol) was added under vigorous stirring in order to remove the acid and to obtain the free amine. After 10 min, the reaction mixture was filtered with a syringe using a 0.45 µm filter in order to remove inorganic salt. To free glucosamine, 0.463 mmol of citral was added at 35 °C and stirred for 10 min, when the formed imine precipitated. The obtained precipitate was recovered and washed with dichloromethane and further dried under vacuum.

### 2.4. Characterization Techniques

Obtaining of the xerogels was realized by freeze-drying of the hydrogels with a LABCONCO FreeZone FreezeDry System (Québec City, QC, Canada) at −54 °C and 1.510 mbar for 24 h. 

The NMR spectra of C2–C4 hydrogels and of MC were recorded with a Bruker Avance DRX 400 MHz Spectrometer (Bruker, Ettlingen, Germany) equipped with a 5 mm QNP direct detection probe and z-gradients. The hydrogels were prepared in deuterium oxide for analysis. 

Fourier-Transform infrared (FTIR) spectra of the xerogels, chitosan and model compound were performed using a Bruker Spectrophotometer (VERTEX 70) by the ATR method from 4000 to 600 cm^−1^. The spectra were processed with OPUS 6.5 software. 

Xerogel morphology was evaluated with a scanning electron microscope SEM EDAX—Quanta 200 (Eindhoven, Germany). The obtained images were processed with Image J Software. 

Wide angle X-ray diffraction was performed on xerogel pellets using a Rigaku Miniflex 600 diffractometer with CuKα-emission in the angular range of 2–40° (2θ), a scanning step of 0.0025° and a recording rate of 1**°**/min.

Polarized optical microscopy images were recorded on xerogels with a Leica DM2500 microscope (Hamburg, Germany). 

Water uptake capacity (WUC) was evaluated on rectangular-shape pieces of xerogels which were previously kept in the oven for 24 h. The experiments were performed in triplicate and the results are presented as the mean value ± standard deviation (SD). By knowing the exact mass of the samples before and after the water uptake experiment, the water uptake capacity of the xerogels was calculated using the following equation: WUC = (Ms − Md)/Md, where Ms is the mass of the xerogel after water absorption and Md is the mass of the xerogel in a dried state.

Xerogel stability was evaluated by immersing pieces of xerogels of 10 mg each into 1 mL deuterium oxide with 10 µL hydrochloric acid and stirred for 24 h. The supernatant was analyzed by ^1^H-NMR spectroscopy, while the insolubilized part was lyophilized and analyzed by FTIR spectroscopy. Further, xerogel stability was also evaluated by determining the mass loss of xerogels in an acetic buffer solution after 1 h and 24 h. Pieces of xerogels with known mass (m_i_) were immersed in acetic acid solution at a concentration of 1 mg/mL. After 1 h and 24 h, respectively, the supernatant was removed and the swollen samples were lyophilized and weighted (m_f_). The mass loss was calculated using the following equation: Mass loss = $\frac{m_{i}-m_{f}}{m_{f}}*100\;\%$. 

The porosity of the xerogels was calculated by a protocol previously reported in the literature [39,40]. Small pieces of xerogels, with a cubic shape, a volume of V_tot_ = l^3^ (cm^3^) and a known mass (m_1_) determined by gravimetric measurements were sunk into cyclohexane, which is a nonsolvent. The pieces of xerogels were removed from time to time and weighed up to reaching equilibrium. When equilibrium was reached, the mass of the xerogel containing cyclohexane was measured (m_2_). The volume of the absorbed cyclohexane into the pores of the xerogels was calculated with the following equation: V_cyclohexane_ = (m_2_ − m_1)/_ρ_cyclohexane._ The volume of the cyclohexane which entered into the pores was equal to the volume of the pores of the xerogels (V_pores_). Thus, the porosity was calculated with the following equation: P = V_pores/_V_tot_ × 100. The experiments were performed in triplicate and the represented values are the average value ± S.D. 

The gel fraction was calculated by a method previously reported in the literature [39]. Pieces of samples of known mass (m_1_) were immersed in 40 mL of bidistilled water for 24 h. After 24 h, the samples were removed and dried in vacuum at 60 °C for 48 h. Further, the samples were weighted and m_2_ was determined. The gel fraction was calculated using the following equation: Gel fraction = m_2_/m_1_.

Mercury recovery ability was investigated on pieces of xerogels in mercury acetate solutions with concentrations of 5 g/L and 10 g/L. Therefore, pieces of xerogels with a mass between 10 and 20 mg (m_1_) were immersed in a mercury acetate solution at a final concentration of 1 mg/mL. After 24 h, the supernatant was removed, and the remaining samples were lyophilized and weighted (m_2_). The mass of recovered mercury was determined by the difference between m_2_ and m_1_. The samples, after being in contact with mercury acetate solution and lyophilization, were analyzed by FTIR, SEM, EDAX and WXRD.

The interference with other metals in the process of mercury retention was evaluated by determining the increase in the mass of C4 xerogel when immersed in a mixture of mercury acetate and other metal acetate solutions. After 24 h, the solid samples were recovered and lyophilized in order to determine the final mass. The total amount in g/g of absorbed metal ions was calculated by the difference between the final mass and the initial mass divided by the initial mass of the xerogel. 

## 3. Results and Discussions

Hydrogels based on chitosan and hydrophobic citral were synthesized by a method previously developed in our group based on three simultaneous processes: (i) the acid condensation between the amino groups of chitosan with the aldehyde group of citral, (ii) the self-ordering of the newly formed imine linkages and (iii) the self-assembling of the newly formed amphiphiles by hydrophobic–hydrophobic interactions between the citral moieties grafted on chitosan backbone [36,39,40,41,42,43] (Scheme 1). By varying the molar ratio between reagent functionalities, three hydrogels were obtained using 4/1, 3/1 and 2/1 molar ratios between amino and aldehyde groups. Having in mind the ability of imine linkages to coordinate metal ions, along with the porous nature of the xerogel, the ability of the corresponding xerogels to be used as materials for the recovery of mercury ions was investigated. 

### 3.1. Structural Characterization by ^1^H-NMR

The hypothesis of imine’s formation between chitosan and citral was investigated using ^1^H-NMR spectroscopy. Moreover, in order to gain a deeper insight into the structural characterization of the hydrogels, a model compound was synthesized by reacting citral with glucosamine, which is the structural unit of chitosan. 

**Figure 1:**
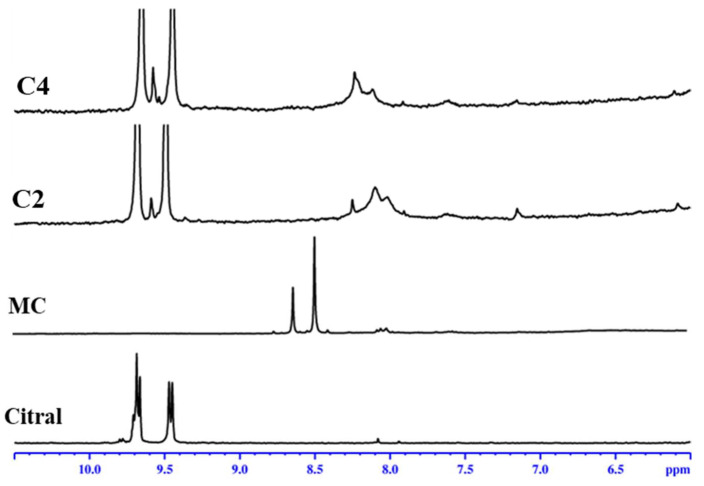
Aromatic region of the ^1^H-NMR spectra of the model compound and two hydrogels.

^1^H-NMR demonstrated the formation of imine linkages between chitosan’s amino groups and the aldehyde group of citral by appearance of the chemical shifts between 8.5 and 8 ppm, corresponding to the protons of the newly formed imine, in sin- and anti-conformations (Figure 1) [38,39]. In the spectrum of the model compound, the signals are clearer and sharper, appearing at 8.6 and 8.5 ppm. Moreover, the spectra of the hydrogels also presented chemical shifts corresponding to aldehyde protons at 9.7 and 9.5 ppm because it is already known and has been demonstrated in our previous studies that the imination of chitosan and citral is an equilibrium process [44,45]. 

### 3.2. Structural Characterization by FTIR

**Figure 2:**
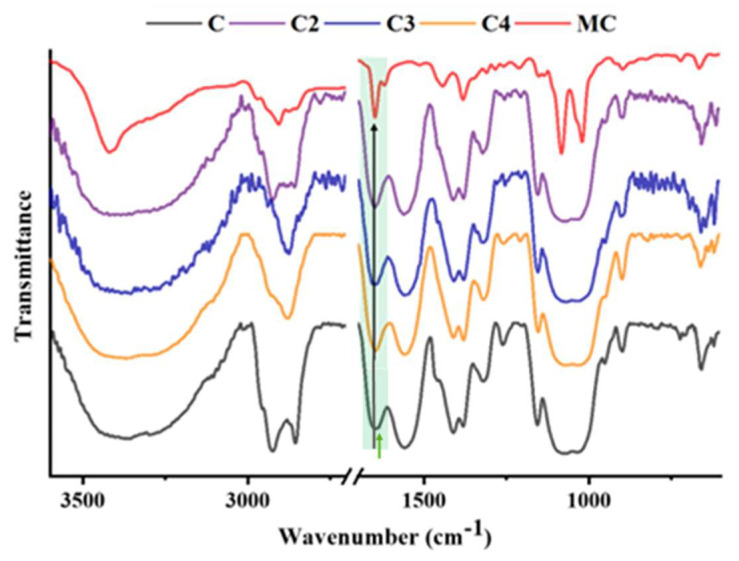
FTIR spectra of chitosan, the xerogels and the model compound (the grey arrow in the spectra marked the presence of imine band around 1645 cm^−1^, while the green arrow indicates the vibration band of the amide group in chitosan).

It is well known that the acid condensation of aldehydes and amines is an equilibrium process in the presence of water. Therefore, in order to evaluate whether the imination reaction was shifted towards products during lyophilization due to the removal of water from the systems, the samples were submitted to FTIR analysis. Moreover, the spectrum of the model compound was also recorded and used for comparison. The formation of the imine linkages between the reagents was confirmed by the appearance of a sharp absorption band at 1645 cm^−1^ in the spectra of MC and also of imino-chitosan xerogels (Figure 2) [38]. The band is shifted towards a higher wavenumber and is sharper than the one corresponding to the vibration of the amide group in chitosan (Figure 2). Moreover, in the spectra of the xerogels, the band corresponding to the unreacted aldehyde is absent, indicating the shifting of the reaction equilibrium towards products. Significant changes were also observed between the 3700 and 2700 cm^−1^ spectral region, the two broad maxima from chitosan being shifted to higher wavenumbers, indicating the modifications of the hydrogen bond environment from the chitosan structure due to imination. This can be explained on one side by the consumption of the amino groups of chitosan in the reaction with citral and on the other side by the changes in terms of intermolecular hydrogen bonds due to the grafting of citral moieties on the chitosan backbone. 

### 3.3. Morphological Characterization by SEM Porosity and Gel Fraction

**Figure 3:**
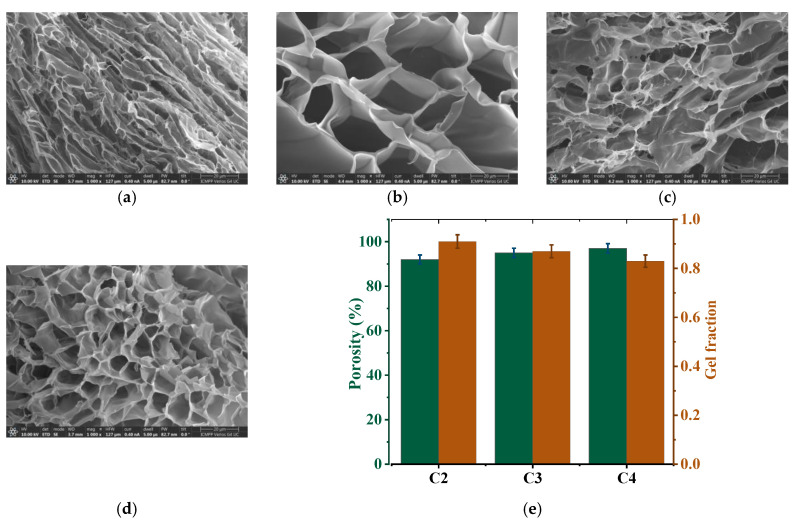
SEM microphotographs of chitosan and the corresponding xerogels (**a**,**d**). Porosity and gel fraction (**e**). (**a**) C; (**b**) C2; (**c**) C3; (**d**) C4.

Xerogel morphology was evaluated by scanning electron microscopy (Figure 3a–d). All samples, including lyophilized chitosan, presented porous morphology, with micrometric pores with sizes in the range of 20–30 µm. No clear trend was observed regarding the dependence between pore size and the amount of citral grafted on the chitosan backbone, similar to previous studies [16]. 

Moreover, the porosity and gel fraction of the xerogels were determined to be important parameters which describe xerogels with an impact on their potential applications. As expected, the porosity of the samples slightly increased with the decrease in the amount of citral grafted on the chitosan backbone, most probably because of the lower density of hydrophobic clusters which crosslink the chitosan chains (Figure 3e). The data obtained from the porosity measurements correlated very well with the values obtained for the gel fraction parameter which represent the “insoluble” part of the hydrogels, which decreased with the decrease in the amount of citral used in hydrogel synthesis. 

### 3.4. Wide-Angle X-ray Diffraction 

**Figure 4:**
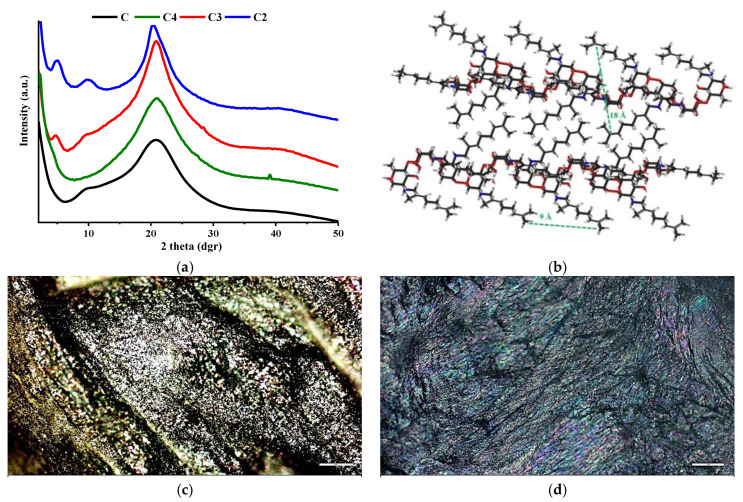

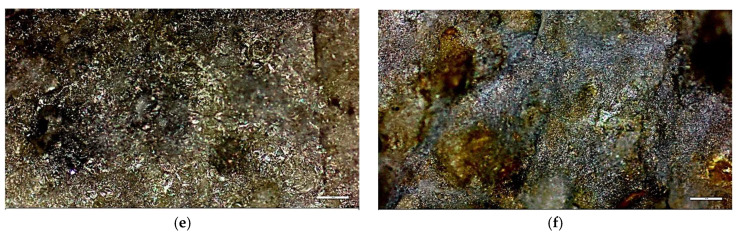
Diffractograms of chitosan and xerogels (**a**). Schematic representation of the xerogels’ supramolecular architecture (**b**) and polarized optical microscopy images of the samples (**c**–**f**) (Scale bar: 100 µm). (**c**) C; (**d**) C2.

Wide-angle X-ray diffraction was used in order to characterize the systems from the supramolecular point of view. Therefore, the chitosan diffractogram presented two reflections at 9.6 and 20.6 two-theta degrees corresponding to the distances of 9.2 and 4.3 Å, respectively, due to its well-known semicrystalline structure (Figure 4a). The grafting of hydrophobic citral moieties on the chitosan’ backbone led to a three-dimensional layered architecture (Figure 4b), which was more evident in the case of the hydrogel prepared using a higher amount of aldehyde (C2) [38]. C2′s diffractogram presented three diffraction peaks corresponding to the following distances: (i) 18.02 Å between the hydrophobic layers, (ii) 9.04 Å between two rigid citral moieties grafted on the chitosan backbone and (iii) the last one of 4.4 Å between two chitosan chains within the same layer. The semicrystalline nature of the xerogels and of chitosan was also supported by polarized optical microscopy images, all samples presenting strong birefringence due to their high degree of ordering at a supramolecular level (Figure 4c–f) [46]. 

### 3.5. The Investigations of Mercury Absorption Capacity of the Xerogels

#### 3.5.1. Gravimetric Measurements—The Water Uptake Capacity of the Xerogels and Stability in the Acidic Medium

**Figure 5:**
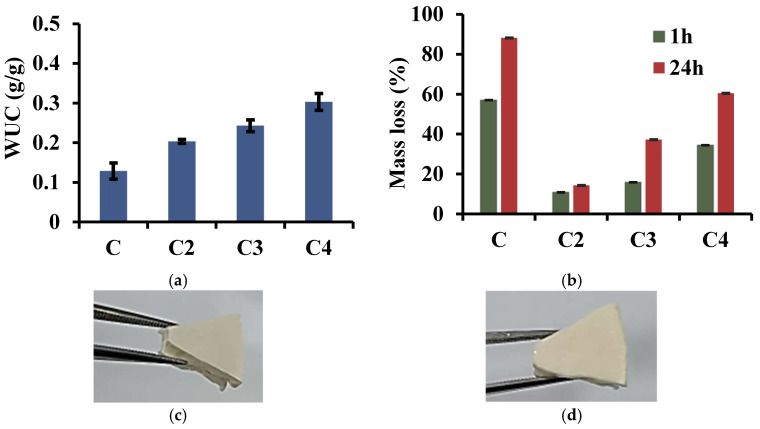

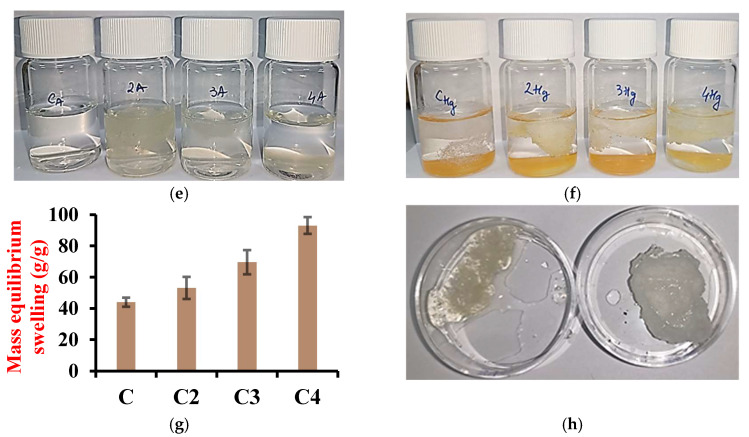
Water uptake capacity of the xerogels (**a**), mass loss in acidic buffer (**b**) and pictures taken during the experiments (**c**) before swelling in water, (**d**) after swelling in water, (**e**) in acetic buffer, (**f**) in mercury acetate solution; swelling degree in mercury acetate solution (**g**) and pictures of C4 in acetate buffer (**left**) and in mercury acetate (**right**) (**h**).

Water uptake capacity (WUC) is an important parameter which influences their performance as materials for the recovery of mercury from waste waters [16]. Therefore, the ability of the xerogels to absorb water was investigated by gravimetric measurements. As expected, this parameter was correlated with the molar ratios between chitosan and citral used in hydrogel synthesis and increased with the decrease in the molar ratio, reaching a maximum of 0.3 g/g for sample C4 (Figure 5). These data correlate very well with xerogel porosity. Moreover, the values for the water uptake capacity are lower than in the case of other systems based on chitosan and monoaldehydes, probably because of the strong hydrophobic character of citral, inducing a strong hydrophobic character in the hydrogels and thus repealing the water molecules. Therefore, in this case, the increase in the mass of the xerogels after immersion in water was more likely caused by the replacement of air from the porous structure of the xerogels with water (Figure 5a). This fact was also confirmed by the visual monitoring of the samples; the xerogels after water absorption did not rehydrate and did not change their shape or volume (Figure 5c,d). 

Taking into consideration the fact that mercury salts generate acidic solutions, the stability of the xerogels and of chitosan was investigated in buffer acetate of pH 3.7 in the presence or absence of mercury acetate. The xerogels behaved differently depending on the molar ratio between chitosan and citral. Therefore, Samples C2 and C3 were the most stable, reforming the hydrogels in 30 min in the acidic solution in the absence of mercury, while Sample C4 swelled very fast and started to disintegrate in 3 h. The C xerogel, due to the absence of crosslinking nodes, started to disintegrate even faster, disappearing in 1 h (Figure 5). In order to quantify the extent of disintegration of the samples in time, even if not visible macroscopically, the samples were lyophilized after being kept for 1 h and 24 h in an acidic medium; mass loss was calculated by gravimetric measurements. Higher values for mass loss were obtained for C and C4 samples, in line with the visual observations of the samples. Mass loss of the C2 sample in the acidic buffer was the lowest, reaching only 14%, due to the higher density of hydrophobic citral clusters. 

Different behavior was observed when the samples were immersed in a mercury acetate solution of 5 g/L, with pH 3.7. The samples swelled in time, without any disintegration observed. The swelling degree of the samples increased with the decrease in the citral content, even hydrogel C4 which fully disintegrated in the acidic buffer becoming rubbery and elastic in the presence of mercury ions (Figure 5). This clearly indicates the ability of mercury ions to strengthen the imino-chitosan hydrogel network due to the formation of new bonds. 

#### 3.5.2. The Stability of the Imine Linkage in an Acidic Aqueous Solution

**Figure 6:**
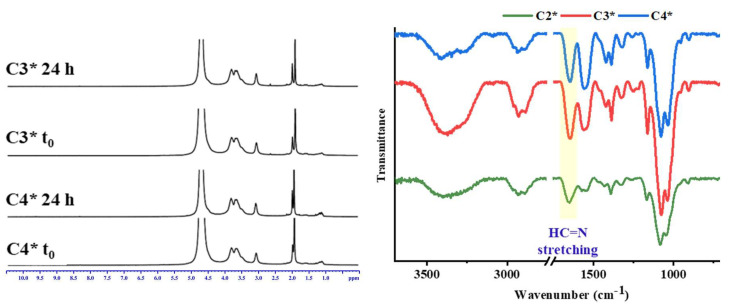
^1^H-NMR and FTIR spectra of the xerogels after being in contact with acidic solution for 24 h.

In order to elucidate why the mass loss in acidic media occurred, the stability of the imine linkage was evaluated in hydrochloric acid with pH 3.7. Therefore, the samples were immersed in deuterium oxide with hydrochloric acid for 24 h, after which the supernatant was analyzed by ^1^H-NMR spectroscopy, while the solid part, the undissolved one, was analyzed by FTIR spectroscopy (Figure 6). In the case of C2 sample, the NMR was not recorded because the deuterium oxide hydrochloric acid solution swelled the sample and no dissolution occurred. 

The ^1^H-NMR spectra of C3 and C4 samples were identical with the one of chitosan, no peaks from the citral or imino-chitosan derivative being observed in the spectra, indicating the stability of the imine linkage in the acidic solution due to the presence of hydrophobic clusters which act as a shield for imine bonds (Figure 6). This was actually confirmed by the FTIR spectra of the undissolved part. In the FTIR spectra of the samples kept for 24 h in the acidic solution, the vibration band corresponding to the group stretching vibration of the imine linkage appeared at 1645 cm^−1^, the spectra of the xerogels identical with the ones of the starting materials, the ones of xerogels without being kept in acidic solution. 

#### 3.5.3. The Evaluation of the Xerogels as Materials for Mercury Recovery

Citryl-imino-chitosan xerogels were designed based on theoretic premises which should lead to materials with high mercury absorption capacity. Therefore, from the structural point of view, the xerogels should enhance mercury recovery by the presence of coordinating moieties such as hydroxylic and amino chitosan groups, along with the imine linkages formed between chitosan and citral, while from the point of view of their properties, the samples were prepared in the form of xerogels, with high porosity and therefore high surface area, which should also facilitate the process of mercury absorption. Stability tests and the preliminary investigation of the samples’ behavior in a mercury acetate solution revealed that the materials present the potential of being used in this sense, the mercury ions presenting a strengthening effect upon citryl-imino-chitosan networks. 

Therefore, the ability of the xerogels to absorb mercury from mercury acetate solutions was investigated. Due to the fact that during the experiments the mercury acetate solutions were unstable, forming an orange precipitate on the vials which, in accordance with the literature, is HgO [16], quantification could not be performed by analyzing the supernatant and was performed instead by gravimetric measurements. 

**Figure 7:**
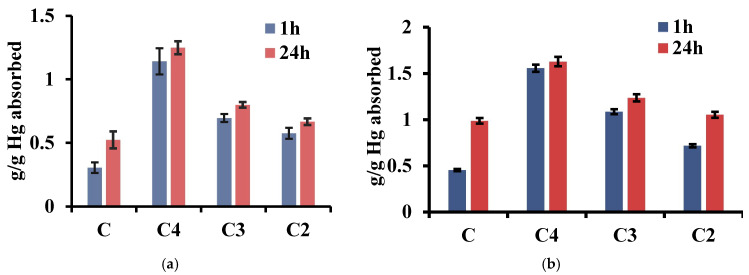
The amount of mercury absorbed by the samples from mercury acetate solutions with two different concentrations. The results represent the mean value of three independent measurements ± S.D. (**a**) From the 5 g/L solution; (**b**) From the 10 g/L solution.

The data showed that mercury recovery is a process which depends on both the crosslinking degree of the xerogels and the concentration of mercury acetate solutions. As expected, a higher uptake capacity was obtained in the case of the more concentrated solution, reaching a maximum of 1.6 g/g for the C4 sample (Figure 7). Very interestingly, the increase in the contact time between the xerogels and the mercury solutions did not lead to a significant increase in recovery efficiency. Moreover, it was observed that among the three samples, the one containing the lowest density of citryl-imine linkages grafted on the chitosan backbone presented the highest absorption ability, indicating the prevalence of the importance of sample porosity in the mercury uptake process in the detriment of imine linkage density.

#### 3.5.4. The Interference with Other Metals in the Process of Mercury Retention 

**Figure 8:**
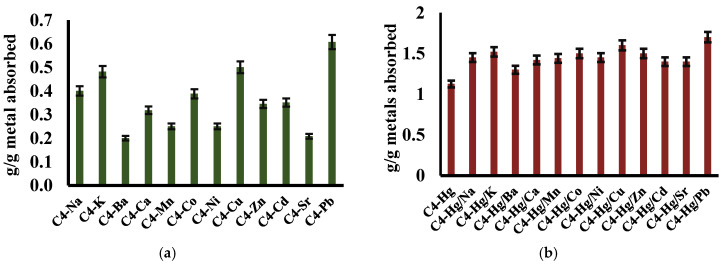
The results of interference experiments—the ability of xerogel C4 to absorb different metal ions from a 5 g/g solution (**a**) and to absorb mercury in the presence of other metal ions (**b**) (5 g/g). The results represent the mean value of three independent measurements ± S.D.

In order to evaluate the interference with other metals in the mercury absorption process from waste waters, the ability of Sample C4 to absorb mercury was evaluated in a mixture of mercury acetate with other metal acetates and compared with the increase in the mass when a pure mercury acetate solution was used. For comparison, the increase in the mass for the C4 sample in the solutions used for the interference tests was also evaluated. Therefore, the C4 xerogel was able to absorb different amounts of metal ions as presented in Figure 8a, with the highest increase in the mass of the C4 sample for lead, copper and potassium. The immersion of the C4 sample in mixtures of mercury acetate with other metal acetates led to higher values of mass increase than the one obtained in mercury acetate only (Figure 8b). Even if it is not possible to discriminate in a clear manner between the absorbed metal ions (how much from the mass increase is due to the mercury and how much is due to the other metal), the high values of the absorbed metals revealed the high potential of the xerogels to be used in metal retention from waste waters.

#### 3.5.5. The Characterization of the Materials after Mercury Recovery

**Figure 9:**
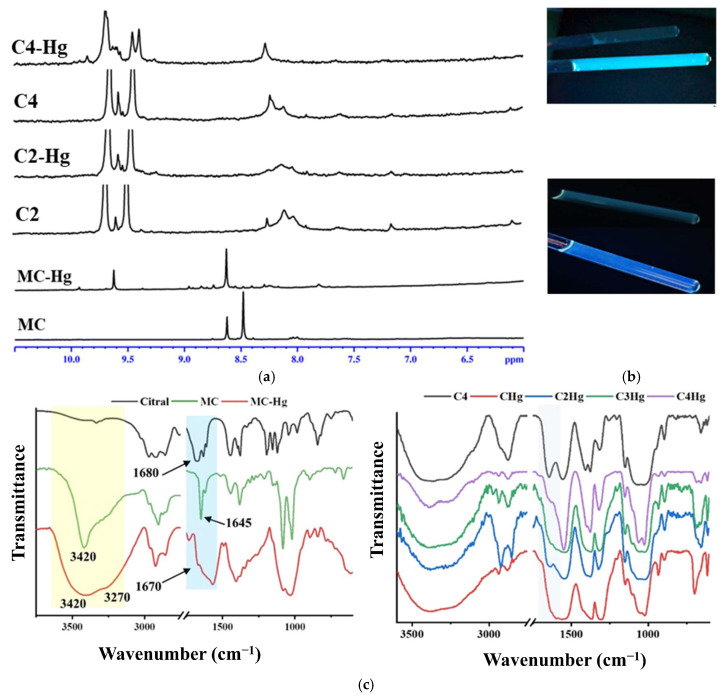
^1^H-NMR spectra of the MC and two hydrogels with and without mercury acetate (**a**), images under UV lamp illumination for C2 (**up**) and MC solution (**down**) (**b**) and FTIR spectra of the samples before and after Hg absorption (**c**).

The recording of the NMR spectra for the hydrogels and for the MC, after being in contact with mercury acetate solution, revealed the disappearing of the signal corresponding to one conformation of the imine proton, indicating its consumption to coordinate the mercury ions or the stabilization of only one imine isomer. By comparing the ratios between the integrals of the aldehyde and imine protons for the samples containing mercury acetate with the one of the samples without mercury acetate, a decrease in the value of the integral was remarked, corresponding to the imine proton, revealing its consumption in the coordination of mercury ions. This behavior was also noticed in the case of MC, confirmed also by the visual monitoring of the samples (Figure 9). The hydrogels and the MC solution presented luminescence under illumination with an UV lamp, which was quenched by the interaction with the mercury acetate solution (Figure 9). 

Analysis by FTIR spectroscopy of the MC and of the xerogels after being in contact with mercury acetate solution revealed the consumption of imine linkages in coordinating mercury ions by the disappearance, in the case of the C4 sample, the one containing the lowest amount of citral, or by changes in terms of band shape in the case of C2 and C3 samples [47,48]. These data correspond with the previous findings from NMR spectroscopy.

**Figure 10:**
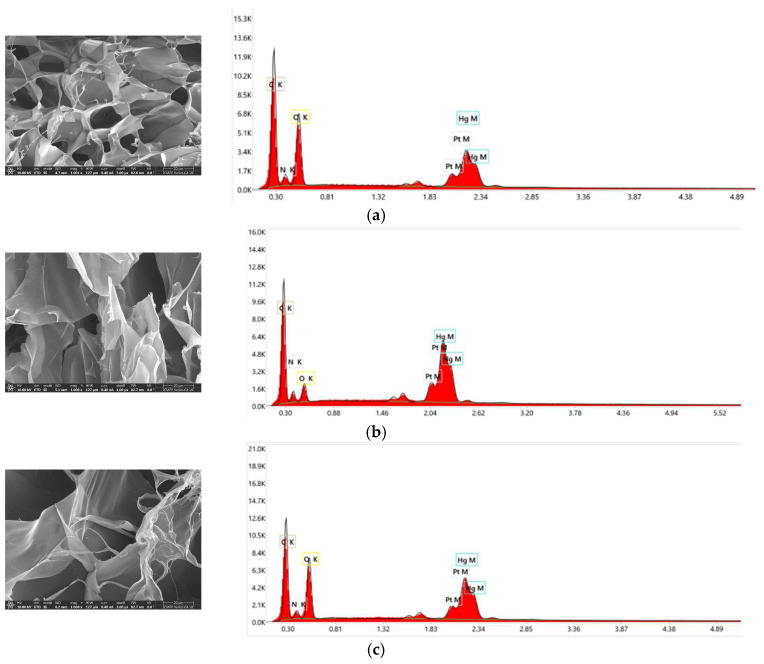
SEM images and EDAX spectra for the xerogels after mercury recovery tests for sample C2 (**a**), C3 (**b**) and C4 (**c**).

The morphological investigations of the xerogels by SEM after being in contact with the mercury acetate solution did not reveal the presence of any mercury acetate crystals on xerogel surfaces or in the pores of the materials, but an increase in the pore walls was observed, the structure becoming more robust. This indicated the predominance of the mercury complexation by the amino, hydroxylic and imine group in the detriment of its physical absorption (Cibotaru et al., 2022 [46]; Ferraz de Paiva et al., 2017 [48]; Sultan et al., 2015 [47]). The quantitative determination of mercury from the samples was evaluated by EDAX. All the samples presented mercury on their surface in different amounts, 31% for sample C2 and 46% for sample C4 (Figure 10). The higher absorption capacity of the C4 sample in comparison with the one of the C2 sample can be explained when considering two different factors: on the one side, from the compositional point of view, sample C2 contains a higher percentage of chitosan reported to the entire mass of the xerogel, ~84%, in comparison with only 72% for sample C2. Taking into consideration that the literature data show that chitosan’s hydroxylic and amine groups can act as complexation moieties for mercury [12,16,22,23], a higher amount of chitosan would lead to a higher recovery capacity for mercury ions. On the other side, there are differences in terms of morphology and the density of the hydrophobic clusters, which is lower in the case of sample C4, conferring better permeability of the mercury acetate solution through the network. 

**Figure 11:**
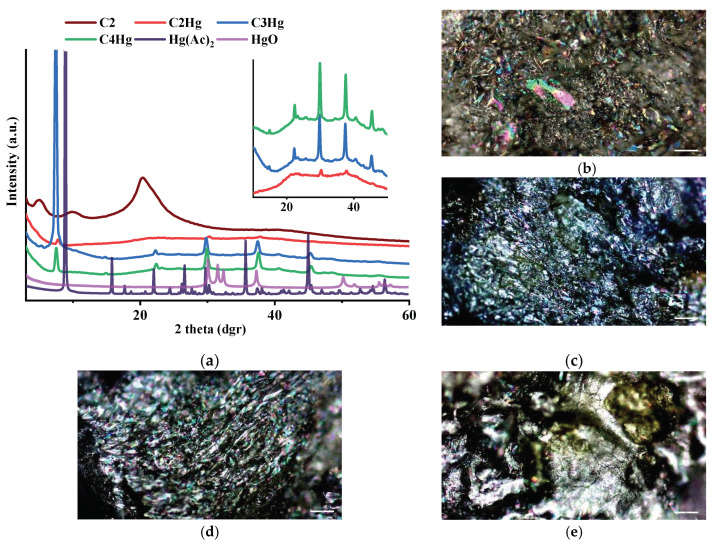
Diffractograms of the xerogels after mercury absorption tests and for HgO and Hg(CH_3_COO)_2_ (**a**) and polarized optical microscopy images of the samples after mercury absorption (**b**–**e**), Scale bar: 100 µm. (**b**) CHg; (**c**) C2Hg; (**d**) C3Hg; (**e**) C4Hg.

The comparison of xerogel diffractograms after mercury absorption tests with the ones of mercury acetate and mercury oxide revealed that the performances of the designed materials are a consequence of their ability to chemically bind mercury ions and not to physically absorb them from the solution, in accordance with the NMR and FTIR spectroscopy measurements and with the lack of crystals on xerogel surfaces as evidenced in SEM images. In xerogel diffractograms, there appears a peak from the mercury acetate diffractogram which is shifted towards a lower 2-theta dgr, from 9 dgr to 7.5 dgr, indicating changes in the supramolecular architecture of the mercury acetate due to the interactions with the xerogels (Figure 11). 

## 4. Conclusions

The study presents the synthesis and characterization of xerogels based on citryl-imino-chitosan derivatives and their use as materials for mercury recovery. The hydrogels were obtained by acid condensation of chitosan and citral, followed by self-assembling of imino-chitosan derivatives by hydrophobic/hydrophobic interactions, forming clusters which act as crosslinking nodes for chitosan backbones, also protecting the formed imine linkages. The structural characterization of the materials by FTIR and NMR spectroscopy, along with morphological and supramolecular characterization, demonstrated the hydrogelation mechanism. The xerogels were highly porous, able to absorb water and rehydrate in the acidic solution, while in contact with the mercury acetate solution they formed rubbery materials able to absorb 1.6 g/g mercury. The structural, morphological and supramolecular characterization of the xerogels after mercury recovery experiments revealed that the mechanism of recovery is mainly based on the formation of new linkages between mercury and xerogel moieties, and not a physical phenomenon, showing that the designed materials can be successfully used as mercury eco-sorbents. 

## Figures and Tables

**Table 1:** Composition of the obtained samples.

Code	NH_2_/CHO Ratio	m_chito_ (mg)	V_H2O_ (mL)	V_CH3COOH_ (µL)	m_ald_ (mg)	V_et_ (mL)	m_dried subs_ (mg)	m_xerogel_ (mg)	Yield (%)
**C**	-	300	15	105	-	-	-	300	100
**C2**	2	300	15	105	116.5	11.6	416.5	416	99.9
**C3**	3	300	15	105	77.6	7.76	377.6	377	99.8
**C4**	4	300	15	105	58.8	5.88	358.8	358	99.8

m_chito_ = the mass of chitosan; V_H20_ = the volume of water used for chitosan solubilization; V_CH3COOH_ = the volume of acetic acid used for chitosan solubilization; m_ald_ = the mass of aldehyde; V_et_ = the volume of ethanol used for aldehyde solubilization; m_dried subs_ = the total mass of reagents; m_xerogel_ = the mass of the hydrogel after lyophilization.

## Data Availability

The data presented in this study are available in the present article.
